# The TGM2 inhibitor cysteamine hydrochloride does not impact corneal epithelial and stromal wound healing *in vitro* and *in vivo*

**DOI:** 10.1016/j.exer.2022.109338

**Published:** 2022-12-05

**Authors:** A.L. Minella, M.I. Casanova, T.J. Chokshi, J. Kang, K. Cosert, M.M. Gragg, M.A. Bowman, M.E. Mccorkell, N.L. Daley, B.C. Leonard, C.J. Murphy, V.K. Raghunathan, S.M. Thomasy

**Affiliations:** a Department of Surgical and Radiological Sciences, School of Veterinary Medicine, University of California, Davis, Davis, CA, USA; b Department of Ophthalmology and Vision Science, School of Medicine, University of California, Davis, Davis, CA, USA; c Department of Basic Sciences, College of Optometry, University of Houston, Houston, TX, USA

**Keywords:** Tissue transglutaminase 2, Cysteamine hydrochloride, Cystaran, Corneal wound healing, Fibrosis, Scarring, Phototherapeutic keratectomy

## Abstract

Corneal wound healing is integral for resolution of corneal disease or for post-operative healing. However, corneal scarring that may occur secondary to this process can significantly impair vision. Tissue transglutaminase 2 (TGM2) inhibition has shown promising antifibrotic effects and thus holds promise to prevent or treat corneal scarring. The commercially available ocular solution for treatment of ocular manifestations of Cystinosis, Cystaran^®^, contains the TGM2 inhibitor cysteamine hydrochloride (CH). The purpose of this study is to assess the safety of CH on corneal epithelial and stromal wounds, its effects on corneal wound healing, and its efficacy against corneal scarring following wounding. Quantitative polymerase chain reaction (qPCR) and immunohistochemistry (IHC) were first used to quantify and localize TGM2 expression in the cornea. Subsequently, *(i)* the *in vitro* effects of CH at 0.163, 1.63, and 16.3 mM on corneal epithelial cell migration was assessed with an epithelial cell migration assay, and *(ii)* the *in vivo* effects of application of 1.63 mM CH on epithelial and stromal wounds was assessed in a rabbit model with ophthalmic examinations, inflammation scoring, color and fluorescein imaging, optical coherence tomography (OCT), and confocal biomicroscopy. Post-mortem assessment of corneal tissue post-stromal wounding included biomechanical characterization (atomic force microscopy (AFM)), histology (H&E staining), and determining incidence of myofibroblasts (immunostaining against α-SMA) in wounded corneal tissue. TGM2 expression was highest in corneal epithelial cells. Application of the TGM2 inhibitor CH did not affect *in vitro* epithelial cell migration at the two lower concentrations tested. At 16.3 mM, decreased cell migration was observed. *In vivo* application of CH at 57 mM was well tolerated and did not adversely affect wound healing. No difference in corneal scarring was found between CH treated and vehicle control eyes. This study shows that the TGM2 inhibitor CH, at the FDA-approved dose, is well tolerated in a rabbit model of corneal wound healing and does not adversely affect epithelial or stromal wound healing. This supports the safe use of this medication in Cystinosis patients with open corneal wounds. CH did not have an effect on corneal scarring in this study, suggesting that Cystaran^®^ administration to patients with corneal wounds is unlikely to decrease corneal fibrosis.

## 1. Introduction

Corneal wound healing requires a complex sequential process of signaling and cellular events involving re-epithelialization and conversion of quiescent keratocytes to fibroblasts then myofibroblasts or KFM transformation. While this transformation is necessary for wound healing, persistence of myofibroblasts in the corneal stroma is associated with haze (Gibson et al., 2013). The resultant opacification of the cornea threatens vision by reducing transparency. Current treatments, including topical steroids and/or mitomycin C, are variably efficacious and have been associated with serious complications and ocular toxicity. Topical steroids can decrease vascularization and fibrosis, however, this comes at the risk of infection, cataract formation, and increased intraocular pressure (Boneham and Collin, 1995; Zhan et al., 1992; Gelatt and Mackay, 1998). Similarly, mitomycin C has been employed to decrease scarring by decreasing the number of fibroblasts that transform into myofibroblasts but has been associated with severe secondary effects including iridocyclitis, cataracts, scleral rupture, and glaucoma (Buss et al., 2010). Novel, more targeted, treatments with decreased side effects that provide improved prevention/resolution of opacification would therefore be of great benefit to patients.

A putative therapeutic to inhibit KFM transformation would focus on preventing cross-linking of stromal proteins in the wound bed, a form of post-translational modification resulting in haze formation. Our laboratory has previously shown that increased stiffness during corneal healing precedes the development of corneal haze, and cross-linking activity has been linked to corneal stiffening (Raghunathan et al., 2017; Raghunath et al., 1999). Inhibition of cross-linking may therefore soften the corneal stroma and provide a druggable target to decrease corneal haze in corneal healing processes. One molecule that has been linked to both KFM transformation and cross-linking, is the enzyme tissue transglutaminase 2 (TGM2), which has been shown to increase transformation of fibroblasts to myofibroblasts and to cross-link the epsilon-(gamma-glutamyl) lysine or (gamma-glutamyl) polyamine bonds of extracellular proteins in the extracellular matrix (Raghunath et al., 1999; Murthy et al., 1991; Olsen et al., 2011). In the eye, studies have shown TGM2 expression in the eyelids, meibomian glands, sclera, and cornea (Barathi et al., 2011). TGM2 expression is higher in some patients with dry eye disease versus normal controls (Aragona et al., 2015). Its expression has also been shown to be elevated in glaucomatous trabecular meshwork TM cells and has been implicated as a contributing factor to glaucoma due to cross-linking of the TM (Tovar-Vidales et al., 2008). Furthermore, knocking out TGM2 in mice with and without ocular hypertension resulted in decreased IOP (Raychaudhuri et al., 2017). Though studies investigating TGM2 in the cornea are limited, TGM2 expression in the rat, mouse, and human cornea has been demonstrated, with a proposed role in epithelial cell adhesion and migration during wound healing (Zhang et al., 2004; Tong et al., 2013). Moreover, TGM2 expression has been demonstrated in human and monkey corneas with demonstrated cross-linking in corneal epithelium and basement membrane (Raghunath et al., 1999).

TGM2 inhibition for the treatment of fibrosis is currently an active area of research in multiple organ systems. TGM2 inhibitors are currently under investigation as treatments for diseases such as pulmonary fibrosis, diabetic nephropathy, Huntington’s, and Celiac disease, with the latter underway in phase 2a clinical trials (Olsen et al., 2014; Huang et al., 2009; Kumar et al., 2015; Rauhavirta et al., 2013). While TGM2 inhibition was shown to be an effective component of a combination therapy against allergic conjunctivitis in a guinea pig model, no studies have been conducted investigating the effect of TGM2 inhibition on corneal wound healing or on the development of cornea haze and scarring (Sohn et al., 2003). The compound cysteamine hydrochloride (CH), a known TGM2 inhibitor, is already available in an FDA-approved topical formulation for the ocular manifestations of Cystinosis (Cystaran^®^ Sigma-Tau Pharmaceuticals, Inc., Gaithersburg, MD, USA), which include corneal ulceration and secondary corneal scarring (Makuloluwa and Shams, 2018). This molecule inhibits transglutaminases by competitive inhibition of the transamidation reactions that transglutaminases catalyze (Jeitner et al., 2018). We sought to determine if treatment with CH could be expanded to include modulation of corneal scarring during wound healing processes, and, to assess its safety of application in times of corneal wound presence. Given that patients with ocular manifestations of Cystinosis are at risk of corneal ulceration, and patients with any inflammatory or ulcerative corneal condition, including Cystinosis, are at risk of secondary fibrosis, it is important to establish safe and effective treatments for corneal fibrosis in the face of corneal wounds. The specific aims of this study were therefore to determine the impact of cysteamine hydrochloride on corneal epithelial and stromal wound healing *in vitro* and *in vivo*.

## 2. Methods

### 2.1. TGM2 inhibitor

An FDA-approved ocular formulation of cysteamine hydrochloride (hereinafter referred to as CH) is commercially available for ocular manifestations of Cystinosis (Cystaran^®^ Sigma-Tau Pharmaceuticals, Inc., Gaithersburg, MD, USA) but has not been tested for its TGM2 inhibitory properties or effects on corneal fibrosis. A non-pharmaceutical grade form of Cystaran^®^ was generated and evaluated, due to lack of availability of the commercially available formulation for purposes of this study. *In vivo* assays were performed at the concentration of Cystaran^®^, 6.5 mg/mL CH, diluted in a balanced salt solution (BSS) vehicle. Benzalkonium chloride was added at the same concentration as Cystaran^®^ (0.1 mg/15 mL of solution) and pH was adjusted with hydrochloric acid and/or sodium hydroxide to the same pH of 4.1–4.5. The solution was prepared in a sterile hood and filter sterilized by passing through a 0.2 μm filter with negative pressure. A range of concentrations were tested to determine *in vitro toxicity*, focusing on concentrations relevant to the commercial formulation (0.163, 1.63, and 16.3 mM). Minimal *in vitro* safety testing was performed here, given the already published literature concerning its safety to gain FDA approval (Huynh et al., 2013; Shams et al., 2014; Makuloluwa and Shams, 2018). For all assays, a vehicle control matching the vehicle without the active ingredient (CH) was utilized consisting of the BSS, and benzalkonium chloride formulated to the same pH as the test solution.

### 2.2. Cell culture

Two cell types were utilized for the below assays. Human telomerase reverse transcriptase-immortalized corneal epithelial (hTCEpi) cells (donated by Dr. James Jester, PhD (University of California, Irvine) were cultured via previously published methods (Makuloluwa and Shams, 2018). Primary rabbit corneal fibroblasts (RCFs) were isolated from freshly enucleated rabbit corneas and cultured as previously described (Dreier et al., 2013; Thomasy et al., 2018).

### 2.3. In vitro TGM2 localization and relative quantification

To determine relative quantification of TGM2 expression across corneal cell types, RNA was extracted using the GeneJET RNA Purification Kit (K0732 Thermo Scientific, Waltham, MA) from hTCEpi cells and rabbit corneal stromal cells treated with and without TGF-β1 (2 ng/mL for 24 h), to represent myofibroblast and fibroblast cell types. RT-PCR was performed using the SensiFAST Probe Hi-ROX One-Step Kit (BIO-77005 Bioline, Taunton, MA) and TaqMan aptamers specific to rabbit TGM2, with glyceraldehyde 3-phosphate dehydrogenase (GAPDH) serving as an endogenous control. Gene expression data was normalized relative to the expression of mRNA from RCFs grown in the absence of TGF-β1 using the ΔΔCt method.

To determine the localization of TGM2 across the cornea, immunohistochemistry (IHC) was performed on 14-μm sections of corneas from New Zealand White rabbits free from signs of corneal disease, fixed with 4% paraformaldehyde, and routinely processed and embedded in paraffin. Sections were compared against a mouse heart positive control and no-antibody negative control; IHC was conducted as previously published (Raghunathan et al., 2017). Briefly, slides were de-paraffinized and then rehydrated. After antigen-retrieval and blocking, the tissue was incubated with a primary antibody specific to TGM2 (#PA5–18728, Invitrogen, Waltham, Massachusetts, USA), followed by incubation with a fluorescent secondary antibody. Slides were then treated with Prolong Gold antifade reagent with DAPI and imaged using a Keyence epifluorescence microscope (BZ-X810, Itasca, IL).

### 2.4. In vitro effect of TGM2 inhibition on corneal epithelial cell migration

*In vitro* cell migration of hTCEpi cells was measured using the Oris^™^ 96-well cell migration assay kit following the manufacturer’s instructions (Platypus Technologies, Madison, WI), as previously described (Makuloluwa and Shams, 2018). Briefly, hTCEpi cells were seeded in the wells of a plate around a stopper placed in the center of each well. The cells were incubated at 37 °C and cultured to full confluence surrounding the stopper. CH was then added at the above-described concentrations at the same time the stopper was removed to allow cells to migrate into the central detection zone for 12 h; Negative (vehicle) and positive controls (cytochalasin D, inhibit migration) were run simultaneously. Cells were fixed with 4% paraformaldehyde for 30 min and nuclei were stained with 4′,6-diamidino-2-phenylindole (DAPI) 1:5000. Cells were fluorescently imaged immediately after staining (Keyence Digital Microscope, Osaka, Japan). The area of the migration was measured using ImageJ software (ImageJ, US National Institutes of Health, Bethesda, MD) and the percentage of migration was calculated relative to the negative control.

### 2.5. In vivo effect of TGM2 inhibition on corneal epithelial wound healing

All animal studies were conducted in accordance with the National Research Council’s Guide for the Care and Use of Laboratory Animals and the Association for Research in Vision and Ophthalmology’s Statement for the Use of Animals in Ophthalmic and Vision Research, and were approved by the University of California, Davis Institutional Animal Care and Use Committee.

Bilateral epithelial wounds were surgically created on the corneas of three adult female New Zealand White rabbits, as previously described (Kim et al., 2021). Briefly, under general anesthesia and a surgical microscope, an 8 mm diameter epithelial wound was created utilizing a circular trephine and corneal epithelium spatula.

Rabbits received four times daily (QID) dosing of CH in the right eye, with the left eye receiving vehicle control at the same dosing interval. CH was applied at a concentration of 57 mM, which corresponds to the Cystaran^®^ concentration of 6.5 mg/mL.

Eyes were examined by a masked examiner and a semi-quantitative pre-clinical ocular toxicology scoring (SPOTS) system was used to document adverse events (Last et al., 2011). Color and fluorescein images were taken twice daily until wounds were healed.

Time to wound healing and SPOTS score in eyes that received CH were compared against eyes that received vehicle control. Data were statistically analyzed using repeated measure ANOVA with post-hoc tests on GraphPad Prism Software (GraphPad Software Inc., San Diego, CA).

### 2.6. In vivo effect of TGM2 inhibitor on corneal stromal wound healing and haze formation

Eight adult female New Zealand White rabbits were used for this study. Both eyes of each animal were experimentally wounded, as previously detailed. Briefly, under a surgical microscope, 8 mm axial epithelial wounds were created as previously described (Kim et al., 2021). Stromal wounds were then created in the center of the epithelial wounds using excimer laser photoablative keratectomy (PTK; NIDEK EC 5000 Excimer Laser System, NIDEK Ltd., Gamagori, Japan) to create wounds of consistent size and depth; 6 mm in diameter and 100 μm in depth.

One eye of each rabbit received CH and the contralateral eye of each rabbit received vehicle control (sterile BSS with 0.1% benzalkonium chloride), with eyes that received CH treatment randomly assigned. The inhibitor and control solutions were administered four times daily (QID) for 28 days in 4 rabbits, and for 42 days in the remaining 4 rabbits. The rabbits received analgesia including systemic meloxicam for 7 days, topical 0.3% ofloxacin ophthalmic solution (Akorn, Buffalo Grove, IL) until the cornea had a negative fluorescein stain, and systemic buprenorphine and topical atropine 1% ophthalmic solution (Akorn, Buffalo Grove, IL) until the animals were no longer painful.

The primary investigator (AM), with residency training in comparative ophthalmology, performed all clinical examinations and was masked to treatment eye. Following wounding, on days 7, 10, 14, 21, 28 (n = 8), 35 (n = 4), and 42 (n = 4) ophthalmic examinations and advanced ocular imaging were performed. An image of the wound was taken with fluorescein photography, ocular signs were documented with the SPOTS system, and stromal haze was quantified with slit lamp biomicroscopy using a scoring system previously established in our laboratory (Eaton et al., 2017). Imaging included digital image capture (pre- and post-fluorescein staining), SD-OCT, and digital slit-lamp biomicroscopy, at all imaging time-points, and *in vivo* confocal biomicroscopy at baseline and final time-points.

Ophthalmic examination and wound imaging allowed for timing and assessment of wound closure. Corneal thickness values were measured from SD-OCT. Additionally, this modality was used to measure the depth and density of stromal haze. Corneal endothelial cell density was determined by performing manual cell counts of these cell types on confocal biomicroscopy images as previously described (Morgan et al., 2014). This was performed in three fields and averaged. These imaging modalities, along with examinations and the color and fluorescein images also allowed for monitoring for side-effects from inhibitor or control solution application.

Following euthanasia, corneal buttons were excised for downstream analyses. Specifically, these corneal buttons were divided for atomic force microscopy (AFM), hematoxylin and eosin (H&E) staining, and immunohistochemistry (IHC).

For atomic force microscopy (AFM), a corneal quarter per eye was mounted as previously described (Morgan et al., 2014). Briefly, the sample is placed in the center of an AFM dish coated with a layer of silicone (Sylgard 527, Down Corning, Midland, MI) and immobilized by a 13 mm Thermanox coverslip (ThermoFisher Scientific, Waltham, MA) with a 2 mm window in the center. The coverslip is adhered to the surface with small droplets of cyanoacrylate glue placed around the periphery of the coverslip. The sample was then submerged in Dulbecco’s PBS (DPBS) and force versus indentation curves were obtained as previously described (Last et al., 2009, 2011, 2012) using an MFP-3D Bio AFM system (Asylum Research, Goleta, CA) and silicon nitride cantilevers (PNP-TR-50, NanoWorld, Neuchâtel, Switzerland) with attached borosilicate microspheres (radius = 2.5 μm, Thermo-Fisher Scientific). Cantilevers were calibrated for the deflection inverse optical lever sensitivity (Defl InvOLS) by indentation in DPBS on glass and then calibrated for the spring constant by the thermal method using the Asylum Research software. Force measurements were made at a scan velocity of 1.98 μm/s and each quarter was measured at 5–7 locations (minimum of 10 locations per eye) in the most central area of the corneal quarters, with 5 curves collected at each location. The elastic modulus was determined by a masked analyst by fitting the data to a Hertz model (McKee et al., 2011). Elastic modulus measurements for each eye were pooled and the significance of variation across treatment groups was assessed.

Sections were cut from the paraffin-embedded quarters for histological assessment. IHC was performed as detailed above, to assess relative keratocytes versus myofibroblasts utilizing a marker against alpha-smooth muscle actin (α-SMA) with DAPI nuclear counterstain, according to previously published methods from our laboratory (Kim et al., 2021). IHC sections were quantitatively assessed by scoring each section as positive or negative for α-SMA and control versus treated sections were compared. H&E staining was performed on additional paraffin sections via standard methods. Inflammation was then scored via a previously reported scoring system for H&E sections (Kim et al., 2021).

### 2.7. Statistical analysis

Numerical data, including wound healing times and stromal haze analysis, was statistically analyzed using repeated measure ANOVA with post-hoc tests (when normally distributed) with *P* values ≤ 0.05 defined as significant. Non-parametric tests were utilized when data were not normally distributed, with the same level of significance. All data were analyzed on GraphPad Prism Software version 9.3.1 for mac (GraphPad Software Inc., San Diego, CA).

## 3. Results

### 3.1. In vitro TGM2 localization and relative quantification

**Fig. 1.**
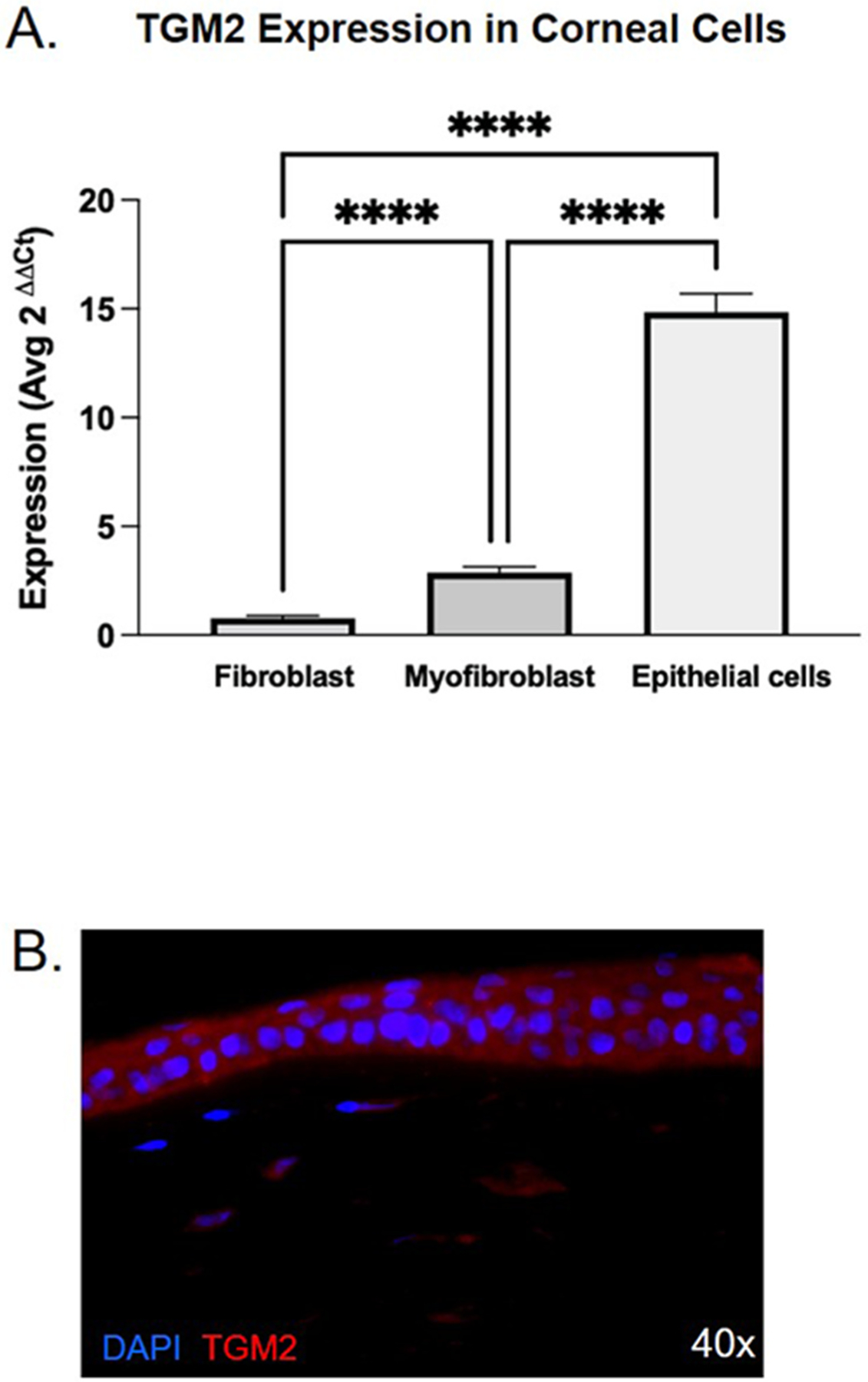
TGM2 is strongly expressed in the cornea during health and healing. A. TGM2 expression by TaqMan qPCR in healthy human corneal cells shows expression in fibroblasts and myofibroblasts, and strong expression in epithelial cells; n=3 per cell type. Samples normalized to GAPDH (**** = *P* < 0.0001). B. Immunohistochemistry shows strong TGM2 expression in the cytoplasm of epithelial cells and lesser expression in stroma in rabbit cornea 7 days post-wounding (n = 3).

The TGM2 mRNA expression was highest in corneal epithelial cells with qPCR and significantly higher mRNA expression than stromal cell types (epithelial cells versus all other cell types: *P* < 0.0001). Myofibroblasts showed a much lower, but the second highest, expression, followed by fibroblasts (myofibroblasts versus epithelial cells and fibroblasts: *P* < 0.0001). IHC corroborated these results in the normal cornea, with high protein expression in the corneal epithelial layer and minimal expression of keratocytes in the corneal stroma (Fig. 1).

### 3.2. In vitro effect of TGM2 inhibition on corneal epithelial wound healing

**Fig. 2.**
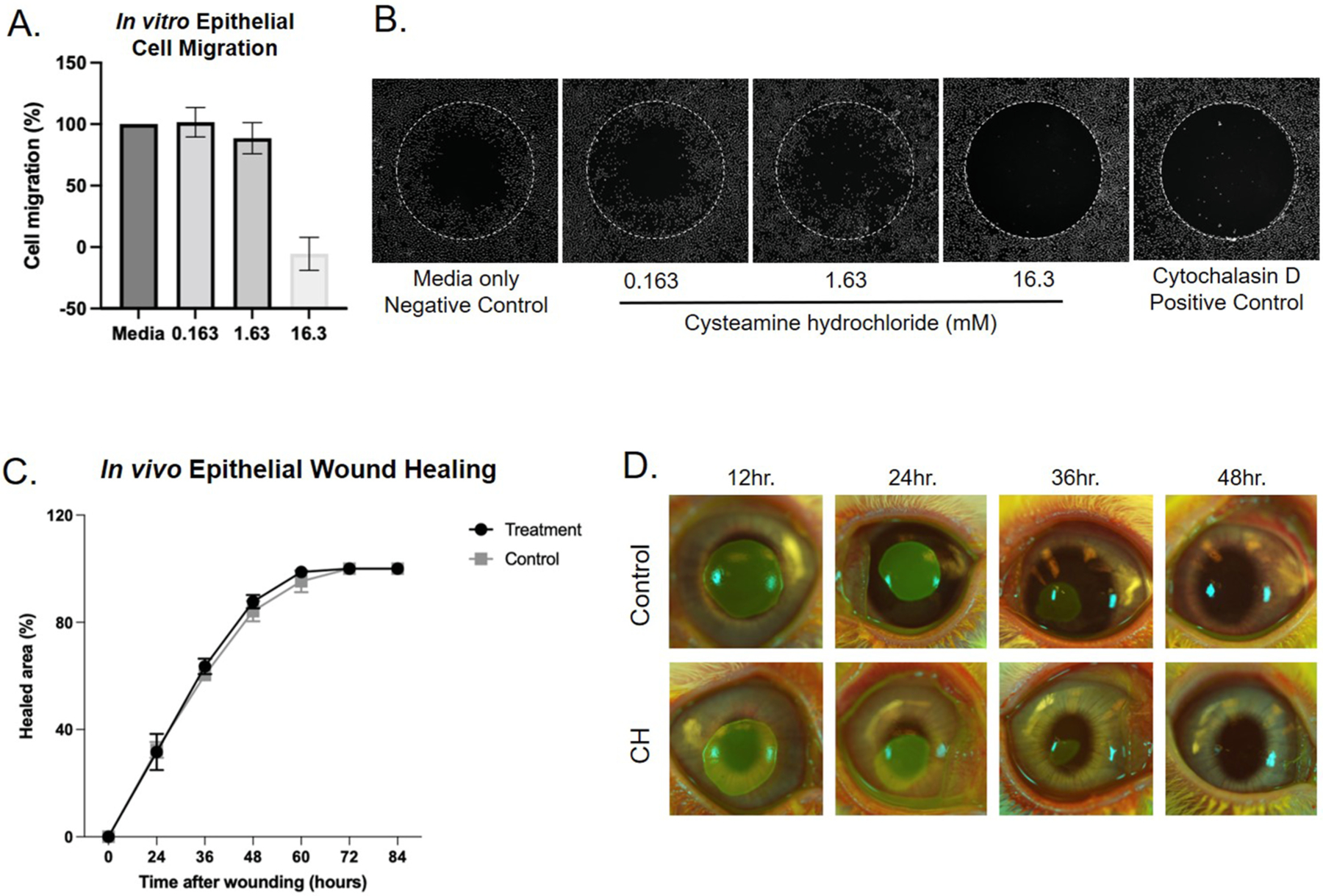
Cysteamine hydrochloride does not adversely affect epithelial cell migration or epithelial wound healing at therapeutic doses. A. In vitro assessment of epithelial cell migration shows cell death at the highest concentration (16.3 mM) but no significant affect on viability or migration at the lowest dose (0.163 mM) or the *in vitro* dose corresponding to the Cystaran therapeutic dose (1.63 mM, *P* > 0.05; Bars = means, error bars = standard deviation). B. Representative images of epithelial cell migration assay showing significantly impaired migration at the highest concentration of CH tested, with no significant difference in migration between low and moderate concentrations and media negative control (n= 3 eyes per group, p > 0.05 for all timepoints). C. *In vivo* epithelial wound healing in a rabbit model was comparable between control and CH treated eyes. D. Representative fluoresceine images of control versus CH treated rabbit eyes illustrating the comparable epithelial healing times. All ulcers healed within 2 days (n = 3 eyes per group).

Epithelial cell migration did not significantly differ between media control and the two intermediate concentrations of CH (0.163 and 1.63 mM with *P* = 0.2199 and 0.3318, respectively). However, the highest concentration of CH (16.3 mM) resulted in cell death and halted cell migration, similar to the cytochalasin D control (*P* < 0.0001) (Fig. 2A and B).

### 3.3. In vivo effect of TGM2 inhibition on corneal epithelial wound healing

There was no difference in healing time of corneal epithelial wounds between rabbit eyes treated with CH versus vehicle control *in vivo* (*P* > 0.05 for all timepoints). Additionally, the *in vivo* SPOTS score did not differ between treated versus vehicle control eyes (*P* > 0.05 for all timepoints) (Fig. 2C and D).

### 3.4. In vivo effect of TGM2 inhibition on corneal stromal wound healing and haze formation

**Fig. 3.**
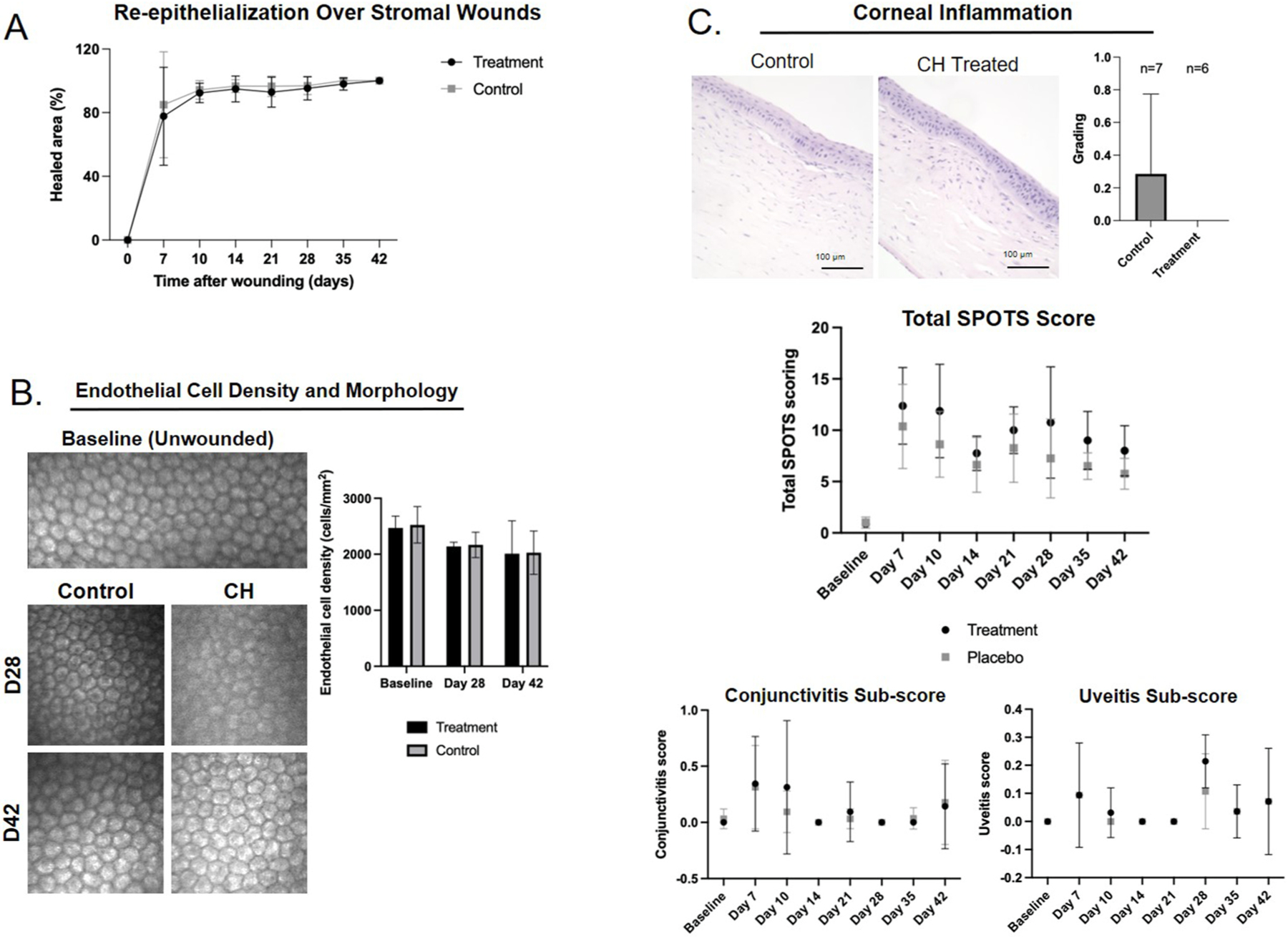
In vivo application of cysteamine hydrochloride at 6.5 mg/mL is safe in a rabbit model of corneal stromal wound healing. A. Application in a rabbit corneal stromal wound model showed no significant effect of CH application on in vivo stromal wound healing (*P* > 0.05 for all timepoints). B. In vivo confocal microscopy showed no toxic effect to corneal endothelial cells with no significant effect on cell density or cellular morphology between treated and control eyes. (*P* = 0.9972 Day 28, *P* = >0.9999 Day 42). C. Assessment of inflammation by scoring of H&E sections as well as by clinical SPOTS scoring, showed no significant difference between CH treated and control eyes. SPOTS score, when divided by extraocular inflammation (conjunctivitis score) and intraocular inflammation (uveitis score), remained insignificant (*P* > 0.05 for all timepoints and scores, n= 8 eyes per group to day 28, n -= 4 eyes per group to day 42).

There was no difference in healing time of rabbit corneal stromal wounds between rabbit eyes treated with CH versus vehicle control *in vivo* (*P* > 0.05 for all timepoints). (Fig. 3A). Confocal microscopy showed that corneal endothelial cell density was not altered by CH treatment (*P* = 0.9972 Day 28, *P* = >0.9999 Day 42), and subjectively, corneal endothelial cells appeared within normal morphological limits for both treated and control eyes. Treatment with CH was well tolerated, as the *in vivo* total SPOTS scores, as well as the conjunctivitis and uveitis sub-scores, did not significantly differ between treated and vehicle control eyes (*P* > 0.05 for all timepoints; Fig. 3C). No appreciable difference in corneal morphology or difference in inflammation scores were detected between treated and vehicle control eyes with H&E staining (*P* = 0.4615; Fig. 3C). Histologic sections showed similar morphological changes in treated versus control eyes, consistent with stromal wounds and subsequent wound healing. These changes consisted of loss of corneal stroma with mild fibrotic changes within the wound bed (Fig. 3C).

**Fig. 4.**
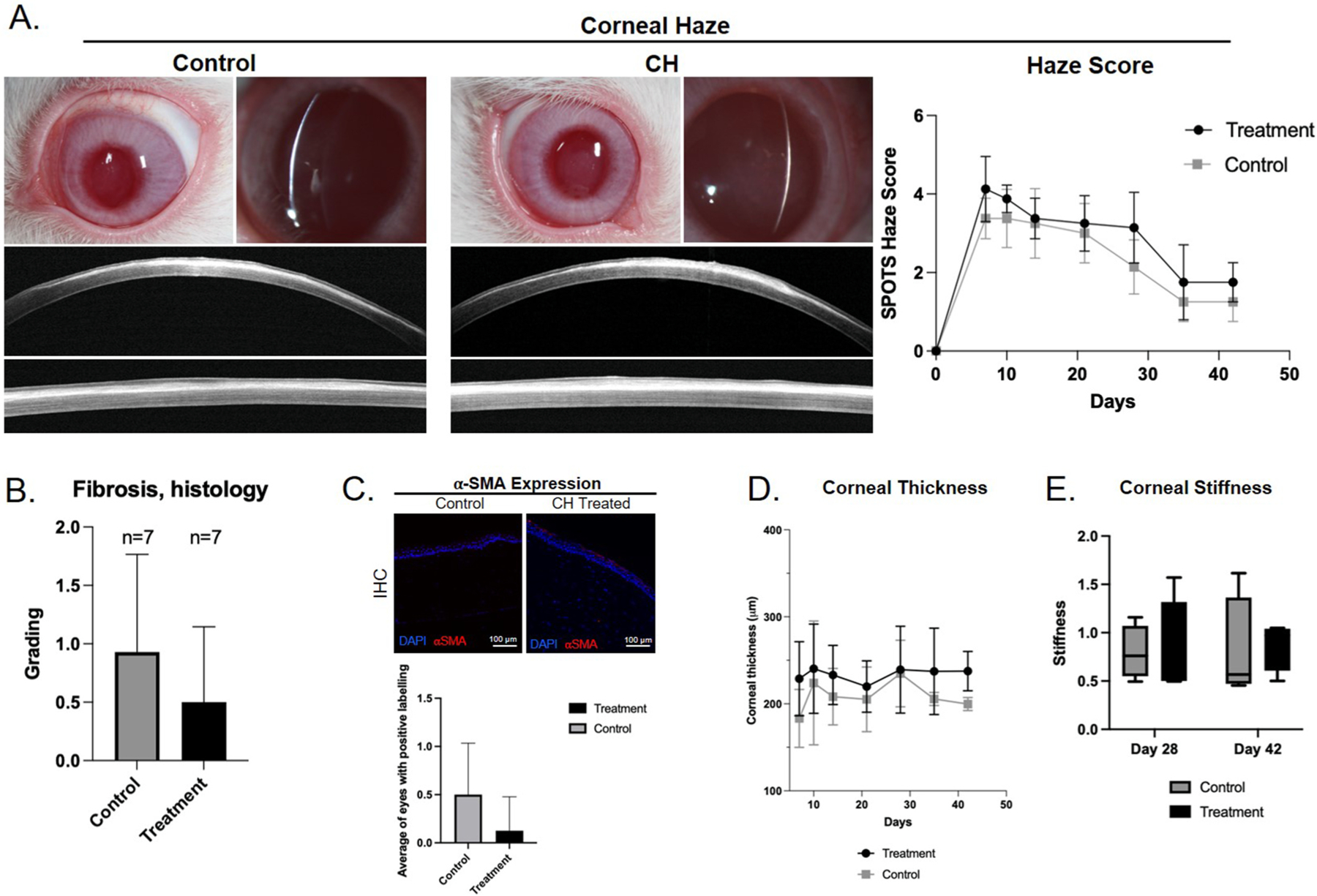
In vivo application of cysteamine hydrochloride at 6.5 mg/mL does not affect corneal scarring. A. Representative clinical, slit beam, and OCT images illustrating the area of corneal haze at final day 42 and graphic representation of clinical haze scores. Corneal haze was present but not significantly different between control and CH treated eyes (n= 8 eyes per group to day 28, n = 4 eyes per group to day 42, *P* > 0.05 for all timepoints). B. Corneal fibrosis, as scored based on H&E histology sections, was not significantly different between treated and control eyes (n = 7 per group, *P* = 0.3473). C. IHC for α-SMA expression showed no difference in expression between treated and control eyes, suggesting no appreciable change in KFM transformation (n = 7 per group, *P* = 0.2821). D. Corneal thickness as measured by OCT, was not different between control and treated eyes suggesting no difference in amount of scarring (n= 8 eyes per group to day 28, n = 4 eyes per group to day 42, p > 0.05 for all timepoints). E. Atomic force microscopy of sections from the area of wounded cornea showed no difference in stiffness between control and treated eyes (n= 4 eyes per time point, *P* = 0.99 at Day 28, *P* = 0.96 at Day 42).

Assessment for an effect on corneal scarring showed no difference between haze score was between CH treated and control eyes (*P* > 0.05 at all timepoints; Fig. 4A). Fibrosis scoring of H&E sections similarly showed no difference (*P* = 0.3473; Fig. 4B). Immunohistochemistry labeling for α-SMA of sections from treated versus control stromal wound beds showed variable α-SMA across all sections, with no consistent subjective or statistically significant difference between treated and control eyes. (*P* = 0.2821; Fig. 4C). Total corneal thickness, as measured by OCT, was not different between treated and control eyes (p > 0.05 for all timepoints; Fig. 4D). Measurement of elastic modulus revealed no difference in stiffness between CH treated and control eyes (*P* = 0.99 for Day 28, *P* = 0.96 for Day 42; Fig. 4E).

## 4. Discussion

TGM2 inhibition is a promising target for a novel therapy against scar tissue formation, as demonstrated in multiple tissues (Olsen et al., 2014; Huang et al., 2009; Kumar et al., 2015; Rauhavirta et al., 2013). Given that the medication Cystaran^®^,which is already FDA-approved for ocular manifestations of Cystinosis and therefore would be faster to market for other conditions, contains as its active ingredient the TGM2 inhibitor cysteamine hydrochloride (CH), we aimed to assess its safety for use in wounded corneas and its potential utility as a medication targeting scar tissue formation following corneal wound healing.

Given that TGM2 has been shown to be integral to epithelial cell adhesion and migration, it was important to first investigate the distribution of TGM2 in the cornea and the safety of CH on corneal epithelial cells (Zhang et al., 2004; Tong et al., 2013). The relatively high prevalence of TGM2 in corneal epithelium was demonstrated here and consistent with previous reports (Barathi et al., 2011; Zhang et al., 2004; Tong et al., 2013). CH adversely affected corneal epithelial cells *in vitro* at a concentration that correlates to a 10-fold increased concentration than that extrapolated from the FDA approved Cystaran^®^ ophthalmic solution concentration (1.63 mM). Inhibition of cell migration was noted at this high *in vitro* concentration on the epithelial cell migration assay. The finding that *in vitro* migration was not affected by the concentration that corresponds to the commercial Cystaran^®^ dosage suggests that Cystaran^®^ is safe to use on epithelial cells and likely represents the highest tolerated dose, above which corneal cell toxicity may be of concern, especially if corneal wounds are present.

Subsequently, CH treatment did not affect corneal epithelial wound healing rates in an *in vivo* rabbit model. These findings have important clinical implications. The results show that application of CH did not clinically affect epithelial wound healing despite its potential as a TGM2 inhibitor and previous reports of the importance of TGM2 in epithelial cell migration and adhesion (Zhang et al., 2004; Tong et al., 2013). This data therefore supports the safety of Cysteamine hydrochloride on corneal epithelial wounds and the continued use of Cystaran^®^ for treatment of corneal cysteine deposits in Cystinosis even when patients develop corneal erosions.

Given the safety shown on epithelial wounds, it was next important to determine the safety and effect of CH application on stromal wounds. Stromal wounds can occur due to corneal injury, infection, or commonly, due to keratorefractive surgeries such as laser-assisted in situ keratomileusis (LASIK) or photorefractive keratectomy (PRK). If corneal scarring develops following these injuries and procedures, devastating visual impairment can occur. Our data showed that application of CH to corneal stromal wounds is safe and did not impair or slow healing of these wounds. Additionally, total clinical SPOTS scoring, including conjunctivitis and uveitis sub-scoring, and H&E analysis showed no difference in inflammatory score between eyes treated with inhibitor versus vehicle control. Lastly, no change in endothelial cell density or morphology was noted, highlighting an important lack of notable endothelial cell toxicity. These data further support the safety of use of Cystaran^®^ for treatment, even when stromal wounds may be present.

Further, these data supported the evaluation of CH against stromal haze and fibrosis given TGM2’s known pro-fibrotic effects, investigating this medication as a potential therapy to treat or prevent this debilitating complication of wound healing. Our results showed no significant difference in corneal haze. There was no difference in alpha smooth muscle actin staining, suggesting no difference in myofibroblast populations in control versus treated eyes. Given that persistence of myofibroblasts in the cornea is correlated with increased haze, this finding suggested that CH application may not decrease haze development. These findings were subsequently supported with our *in vivo* findings that showed no effect of CH application on haze development in a rabbit model, as evidenced by clinical haze scoring, corneal OCT imaging, and H&E sections. Additionally, AFM analysis showed no significant difference in corneal stiffness between eyes treated with CH versus vehicle control. A potential limitation of this study is highlighted in this data - rabbit corneas are markedly softer than those of many other species, particularly humans. This difference in stiffness may make AFM data more challenging to interpret in this model as subtle differences in stiffness, such as may be seen when subtly softening the cornea with an anti-fibrotic treatment, may be more difficult to detect in the rabbit cornea. It is also important to note that while CH has known TGM2 inhibitory properties, TGM2 inhibition was not directly measured in this study. The direct effect of TGM2 inhibition on corneal wound healing and scar formation therefore warrants further investigation in future studies.

## 5. Conclusions

In conclusion, while the highest *in vitro* CH concentration tested inhibited cell migration, application of CH did not affect *in vitro* epithelial cell migration at the two lower concentrations tested, which includes the dose extrapolated from the *in vivo* concentration of Cystaran^®^. *In vivo* application of CH at the Cystaran^®^ concentration of 57 mM (corresponding to the clinical concentration of Cystaran^®^, 6.5 mg/mL) did not adversely affect corneal epithelial or stromal wound healing. This study supports the currently used concentration of CH employed in the FDA approved medication Cystaran^®^. No difference in corneal scarring was found between CH treated and vehicle control eyes.

This study therefore shows that the TGM2 inhibitor CH, at the FDA-approved dose, is well tolerated in a rabbit model of corneal wound healing and does not adversely affect epithelial or stromal wound healing. This supports the safe use of this medication in Cystinosis patients with open corneal wounds. CH did not have an effect on corneal scarring in this rabbit model.

## Data Availability

Data will be made available on request.

## Abbreviations

TGM2: Tissue transglutaminase 2
CH: Cysteamine hydrochloride

## References

[R1] Aragona P, Aguennouz M, Rania L, Postorino E, Sommario MS, Roszkowska AM, , 2015. Matrix metalloproteinase 9 and transglutaminase 2 expression at the ocular surface in patients with different forms of dry eye disease. Ophthalmology 122 (1), 62–71. 25240629 10.1016/j.ophtha.2014.07.048

[R2] Barathi VA, Weon SR, Tan QS, Lin KJ, Tong L, Beuerman RW, 2011. Transglutaminases (TGs) in ocular and periocular tissues: effect of muscarinic agents on TGs in scleral fibroblasts. PLoS One 6 (4), e18326. 21494676 10.1371/journal.pone.0018326 PMC3071819

[R3] Boneham GC, Collin HB, 1995. Steroid inhibition of limbal blood and lymphatic vascular cell growth. Curr. Eye Res 14 (1), 1–10. 7720400 10.3109/02713689508999908

[R4] Buss DG, Sharma A, Giuliano EA, Mohan RR, 2010. Efficacy and safety of mitomycin C as an agent to treat corneal scarring in horses using an in vitro model. Vet. Ophthalmol 13 (4), 211–218. 20618797 10.1111/j.1463-5224.2010.00782.x PMC2904635

[R5] Dreier B, Thomasy SM, Mendonsa R, Raghunathan VK, Russell P, Murphy CJ, 2013. Substratum compliance modulates corneal fibroblast to myofibroblast transformation. Invest. Ophthalmol. Vis. Sci 54 (8), 5901–5907. 23860754 10.1167/iovs.12-11575 PMC3757908

[R6] Eaton JS, Miller PE, Bentley E, Thomasy SM, Murphy CJ, 2017. The SPOTS system: an ocular scoring system optimized for use in modern preclinical drug development and toxicology. J. Ocul. Pharmacol. Therapeut 33 (10), 718–734. 10.1089/jop.2017.0108 29239680

[R7] Gelatt KN, Mackay EO, 1998. The ocular hypertensive effects of topical 0.1% dexamethasone in beagles with inherited glaucoma. J. Ocul. Pharmacol. Therapeut 14 (1), 57–66. 10.1089/jop.1998.14.57 9493783

[R8] Gibson DJ, Tuli SS, Schultz GS, 2013. The progression of haze formation in rabbit corneas following phototherapeutic keratectomy. Invest. Ophthalmol. Vis. Sci 54 (7), 4776–4781. 23800768 10.1167/iovs.13-11976 PMC3719447

[R9] Huang L, Haylor JL, Hau Z, Jones RA, Vickers ME, Wagner B, , 2009. Transglutaminase inhibition ameliorates experimental diabetic nephropathy. Kidney Int 76 (4), 383–394. 19553913 10.1038/ki.2009.230

[R10] Huynh N, Gahl WA, Bishop RJ, 2013. Cysteamine ophthalmic solution 0.44% for the treatment of corneal cystine crystals in cystinosis. Expet Rev. Ophthalmol 8 (4), 341–345.

[R11] Jeitner TM, Pinot JT, Cooper JL, 2018. Cystamine and cysteamine as inhibitors of transglutaminase activity in vivo. Biosci. Rep 38 (5), 1–7. 10.1042/BSR20180691 PMC6123069 30054429

[R12] Kim S, Gates BL, Change M, Pinkerton KE, Van Winkle L, Murphy CJ, , 2021. Transcorneal delivery of topically applied silver nanoparticles does not delay epithelial wound healing. NanoImpact 24, 1–10. 10.1016/j.impact.2021.100352 PMC13531338 35559825

[R13] Kumar A, Singh S, Kumar V, Kumar D, Agarwal S, Rana MK, 2015. Huntington’s disease: an update of therapeutic strategies. Gene 556 (2), 91–97. 25447911 10.1016/j.gene.2014.11.022

[R14] Last JA, Liliensiek SJ, Nealey PF, Murphy CJ, 2009. Determining the mechanical properties of human corneal basement membranes with atomic force microscopy. J. Struct. Biol 167 (1), 19–24. 19341800 10.1016/j.jsb.2009.03.012 PMC2747323

[R15] Last JA, Pan T, Ding Y, Reilly CM, Keller K, Acott TS, , 2011. Elastic modulus determination of normal and glaucomatous human trabecular meshwork. Invest. Ophthalmol. Vis. Sci 52 (5), 2147–2152. 21220561 10.1167/iovs.10-6342 PMC3080174

[R16] Last JA, Thomasy SM, Croasdale CR, Russell P, Murphy CJ, 2012. Compliance profile of the human cornea as measured by atomic force microscopy. Micron 43 (12), 1293–1298. 22421334 10.1016/j.micron.2012.02.014 PMC3622051

[R17] Makuloluwa AK, Shams F, 2018. Cysteamine hydrochloride eye drop solution for the treatment of corneal cystine crystal deposits in patients with cystinosis: an evidence-based review. Clin. Ophthalmol 12, 227–236. 29416314 10.2147/OPTH.S133516 PMC5789046

[R18] McKee CT, Last JA, Russell P, Murphy CJ, 2011. Indentation versus tensile measurements of Young’s modulus for soft biological tissues. Tissue Eng. B Rev 17 (3), 155–164. 10.1089/ten.teb.2010.0520 PMC3099446 21303220

[R19] Morgan JT, Raghunathan VK, Thomasy SM, Murphy CJ, Russell P, 2014. Robust and artifact-free mounting of tissue samples for atomic force microscopy. Biotechniques 56 (1), 40–42. 24447138 10.2144/000114126 PMC4322673

[R20] Murthy SN, Wilson J, Guy SL, Lorand L, 1991. Intramolecular crosslinking of monomeric fibrinogen by tissue transglutaminase. Proc. Natl. Acad. Sci. U. S. A 88 (23), 10601–10604. 1683705 10.1073/pnas.88.23.10601 PMC52977

[R21] Olsen KC, Epa AP, Kulkarni AA, Kottmann RM, McCarthy CE, Johnson GV, , 2014. Inhibition of transglutaminase 2, a novel target for pulmonary fibrosis, by two small electrophilic molecules. Am. J. Respir. Cell Mol. Biol 50 (4), 737–747. 24175906 10.1165/rcmb.2013-0092OC PMC4068920

[R22] Olsen KC, Sapinoro RE, Kottmann RM, Kulkarni AA, Lismaa SE, Johnson GV, , 2011. Transglutaminase 2 and its role in pulmonary fibrosis. Am. J. Respir. Crit. Care Med 184 (6), 699–707. 21700912 10.1164/rccm.201101-0013OC PMC3208598

[R23] Raghunath M, Cankay R, Kubitscheck U, Fauteck JD, Mayne R, Aeschlimann D, , 1999. Transglutaminase activity in the eye: cross-linking in epithelia and connective tissue structures. Invest. Ophthalmol. Vis. Sci 40 (12), 2780–2787. 10549636

[R24] Raghunathan VK, Thomasy SM, Strom P, Yanez-Soto B, Garland SP, Sermeno J, , 2017. Tissue and cellular biomechanics during corneal wound injury and repair. Acta Biomater. 58, 291–301. 28559158 10.1016/j.actbio.2017.05.051 PMC5560898

[R25] Rauhavirta T, Oittinen M, Kivisto R, Mannisto PT, Garcia-Horsman JA, Wang Z, , 2013. Are transglutaminase 2 inhibitors able to reduce gliadin-induced toxicity related to celiac disease? A proof-of-concept study. J. Clin. Immunol 33 (1), 134–142. 22878839 10.1007/s10875-012-9745-5

[R26] Raychaudhuri U, Millar JC, Clark AF, 2017. Tissue transglutaminase elevates intraocular pressure in mice. Invest. Ophthalmol. Vis. Sci 58 (14), 6197–6211. 29222550 10.1167/iovs.17-22236

[R27] Shams F, Livingstone I, Oladiwura D, Ramaesh K, 2014. Treatment of corneal cystine crystal accumulation in patients with cystinosis. Clin. Ophthalmol 8, 2077–2084. 25336909 10.2147/OPTH.S36626 PMC4199850

[R28] Sohn J, Kim TI, Yoon YH, Kim JY, Kim SY, 2003. Novel transglutaminase inhibitors reverse the inflammation of allergic conjunctivitis. J. Clin. Invest 111 (1), 121–128. 12511595 10.1172/JCI15937 PMC151832

[R29] Thomasy SM, Raghunathan VK, Miyagi H, Evashenk AT, Sermeno JC, Tripp GK, , 2018. Latrunculin B and substratum stiffness regulate corneal fibroblast to myofibroblast transformation. Exp. Eye Res 170, 101–107. 29421383 10.1016/j.exer.2018.02.003 PMC5924616

[R30] Tong L, Png E, Aihua H, Yong SS, Yeo HL, Riau A, , 2013. Molecular mechanism of transglutaminase-2 in corneal epithelial migration and adhesion. Biochim. Biophys. Acta 1833 (6), 1304–1315. 23466867 10.1016/j.bbamcr.2013.02.030

[R31] Tovar-Vidales T, Roque R, Clark AF, Wordinger RJ, 2008. Tissue transglutaminase expression and activity in normal and glaucomatous human trabecular meshwork cells and tissues. Invest. Ophthalmol. Vis. Sci 49 (2), 622–628. 18235007 10.1167/iovs.07-0835 PMC2648869

[R32] Zhan GL, Miranda OC, Bito LZ, 1992. Steroid glaucoma: corticosteroid-induced ocular hypertension in cats. Exp. Eye Res 54 (2), 211–218. 1559550 10.1016/s0014-4835(05)80210-6

[R33] Zhang W, Shiraishi A, Suzuki A, Zheng X, Kodama T, Ohashi Y, 2004. Expression and distribution of tissue transglutaminase in normal and injured rat cornea. Curr. Eye Res 28 (1), 37–45. 14704912 10.1076/ceyr.28.1.37.23493

